# Corneal crosslinking with genipin, comparison with UV-Riboflavin in ex-vivo model

**Published:** 2012-04-27

**Authors:** Marcel Y. Avila, Vanessa A. Gerena, Jose L. Navia

**Affiliations:** Department of Ophthalmology, Facultad de Medicina, Universidad Nacional de Colombia, Bogota DC, Colombia

## Abstract

**Purpose:**

To investigate the efficacy and safety of Genipin and UV-riboflavin crosslinking (UV-CLX) in corneal crosslinking.

**Methods:**

Porcine eyes were separated in groups for each crosslinker, genipin 0.25% UV-CLX (clinical crosslinking procedure), glutaraldehyde 0.1% (gold standard crosslinker), and control eyes. Intraocular pressure (IOP) was continuosly monitored by a pressure sensor cannulated to the anterior chamber and the volume was changed. The changes in ocular pressure as a function of change of the ocular volume were evaluated. Ocular rigidity was calculated as the exponential of polynomial quadratic fit. Endothelial damage was evaluated in a viability assay with alizarin red staining as the changes in cell counts.

**Results:**

Significant changes in IOP were observed in the globes were the cornea was stiffened with genipin and UV-CLX treatment (volume 200 μl: Genipin 19.4 mmHg, UVCRX 18.8 mmHg, glutaraldehide 23.9 mmHg, versus control 14.7 mmHg, and 400 μl genipin 31.5 mmHg, UV-CLX 26.0 mmHg, glutaraldehide 37.3 mmHg versus control 18.7 mmHg). The mean ocular ridigity coefficient was genipin 0.0078 mmHg/μl, UV-CLX 0.0065 mmHg/μl, glutaraldehide 0.0092 mmHg/μl, and 0.0046 mmHg/μl for control eyes. Endothelial cell damage was 5.9±1.8% (control), 10.3±1.7% (UV-CLX), 9.4±1.5% (Genipin 0.25%), and 40.1±6.2% (glutaraldehide). Some granules were observed in the UV-CLX group. Reduction of keratocites was observed in the UV CRX crosslinking.

**Conclusions:**

Corneal crosslinking was similar between UV-CLX and genipin with minimal toxicity to endothelial cells. Stiffened corneas by any method induced substancially higher IOP elevation when ocular volume is increased.

## Introduction

Collagen crosslinking has emerged as a method for the treatment of corneal ectasia [1]. In the clinical setting UV riboflavin (UV-CLX) [2] has become as the standard in the treatment of keratoconic eyes, however UV-CLX implies corneal and eye UV irradiation so others systems have been proposed (Genipin, nitroalcohols) [3,4]. Genipin, the active molecule derived from *Gardenia jazminoides*, has been proposed as a alternative crosslinking method showing an increase in the biomechanical of the cornea in vitro and increase of the resistance of the cornea to enzymatic degradation [3], since UV is not required one could consider that the toxicity is lower. We conducted this study to compare genipin with UV-CLX and we evaluated cytotoxic effects in endothelial cells and keratocytes of both treatments in a ex vivo model.

Actual methods of calculating increase in stiffness are based in tensile stress tests, this methods implies cutting the tissue from the sclera and measuring forces in a unidirectional direction, thus the stresses are not distributed in several directions, as in vivo, also the tissue is mounted and the ends of the sample can induce some deformation altering the results, so a method to measure must have to count with all this difficulties, especially when treatments are compared [5]. Whole globe pressure tests overcomes this difficulties and methodology like this, have been used to evaluate ocular rigidity and corneal stiffness. To evaluate the effect of different cross linkers, the relationship between IOP/volume is calculated and the ocular rigidity is determinated [6], so when the cornea is crosslinked the rigidity is increased and this parameter can be calculated in a physiologic range.

Friedenwald [7] introduced the concept of ocular rigidity to determinate the relationship between volume and pressure, this relation gives a estimate of the resistance to the eye to the distensibility, thus if the eye is stiffened the resistance is higher. In this study the biomechanical effects of genipin were compared with UV- CLX and with glutaraldehyde for crosslinking, and the effects in the endothelium were also evaluated in a whole model eye.

## Methods

### Eye preparation

Eighty enucleated porcine eyes with clear corneas were obtained from the local abattoir within 8 to 10 h post-mortem.

### Crosslinking methods

#### Genipin crosslinking

Genipin is disolved in a isotonic medium (PBS) with a customized vehicle at a 0.25% of genipin, then was applied as a droplet every 5 min to a porcine eye in the corneal surface without epithelium (n=20) for 20 min.

#### UV-CLX

Cornea epithelium (n=20) was removed by scraping the corneal surface and then the cornea is impregnated with a solution containing 0.1% riboflavin in 20% dextran T500 (Medio Cross, Kiel, Denmark) for 30 min, followed by 20 min of UVA exposure (370 nm, 3mW/cm^2^) obtained with 2 LEDs (Nitride semiconductors, Tokushima, Japan), a drop is applied every 5 min during the exposure.

#### Glutaraldehyde crosslinking

Glutaraldehyde at 0.1% was applied in the corneal surface for 20 min as a droplet every 5 min and then were washed with saline solution for 15 min (n=20)

#### Controls eyes

Whole fresh eyes were canulated for the pressure measurements (n=20).

### Pressure measurements

**Figure 1:**
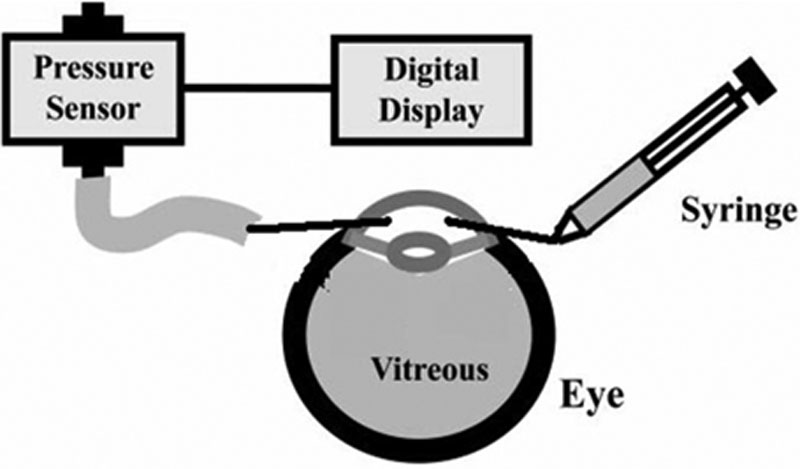
Schematic of IOP –volume evaluation in porcine eyes, a double cannulation of examined eye is done. One cannula is connected to the pressure transducer and the second one is cannulated to the injector.

**Figure 2:**
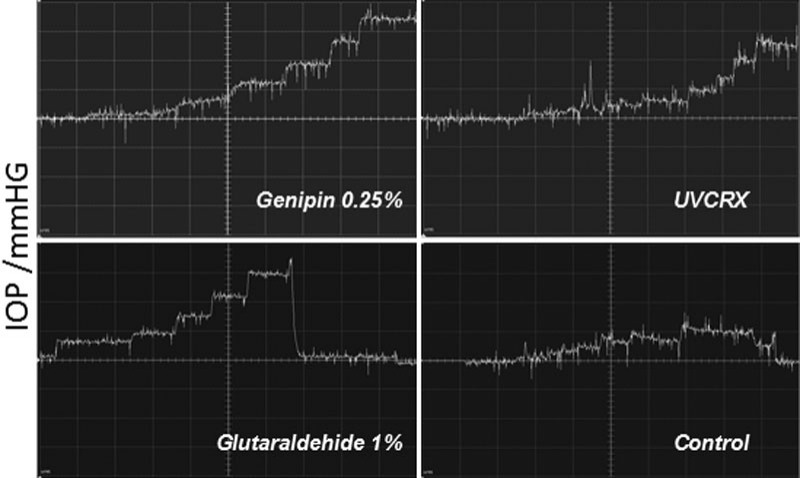
Representative traces of IOP Changes in treated eyes with different crosslinkers. Every vertical square correspond to 5 mmHg.

Eyes were canulated in the anterior chamber, one needle was conected to the infussion system with BSS and the other needle was conected to a pressure transductor connected to a digital converter signal, and a trace of the pressure is obtained (Figure 1). Volume was injected with a pressure pump until a pressure of 10 mmHg is achieved and then 200 μl of volume steps were injected every 5 min. The pressure was monitored continuously (Figure 2). Then the relationship between volume and pressure is obtained and the ocular rigidity is calculated as the exponential of quadratic fit.

#### Endothelial evaluation

A diferent set of treated corneas (n=14 per group) were dissected, carefully mounting the whole eye in a artificial chamber with the cornea upper, then the stroma was carefully dissected with a crescent blade and the Descemet membrane was isolated and transfered carefully to a microscope slide and then was stained with red alizarin at 0.2% in BSS for 2 min and then was washed carefull with BSS, viscoelastic (hyaluronic acid 2%) was applied to the endothelial surface to avoid mechanical endothelial damage then a coverslip was applied. Preparation was mounted in a microscope and photographed, cells were counted using Imagej NIH sofware, celular damage was calculated from the percent of cells with morphological abnormalities or cell loss, also cellular area and the number of cells by mm^2^ were calculated.

#### Stromal and corneal keratocytes

A set of treated corneal stromas treated with the different crosslinkers were fixed with 10% formaledhyde and processed for optical microscopy; keratocytes were counted by field.

### Statistical analysis

In most instances, the statistical significance of comparisons was established with a paired Student’s *t*-test. Ocular rigidity, endothelial cells counts were compared by one-way ANOVA (ANOVA). Statistically significant comparisons were established by the Kruskal–Wallis test using SigmaStat (Systat Software Chicago, IL).

## Results

### Pressure-volume measurements

Increase of the volume in porcine eyes led to a increase in IOP, in crosslinked corneas higher IOP is observed when the volume is increased volume. At 200 µl of volume the eyes with genipin had an IOP 19.3±1.3 mmHg, UV-CLX 18.8±1.25 mmHg, glutaraldehyde 23.9±1.4 mmHg, and controls14.7±1.4 mmHg (p<0.001), At 400 µl IOP was genipin 30.0±1.8 mmHg, UV-CLX 26.05±2.3 mmHg, glutaraldehyde 37.4±2.2 mmHg, and controls 18.7±2.19 mmHg (p<0.001). Figure 2 shows typical traces of eyes treated with diferents crosslinkings.

**Figure 3:**
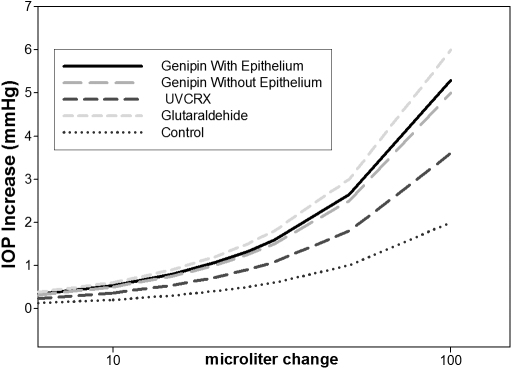
Relationship pressure-volume between different crosslinkers.

Ocular rigidity calculated as the ratio of exponential fit between IOP and volume according to the Friedenwald equation was genipin 0.0078 mmHg/μl, UVCRX 0.0065 mmHg/μl, glutaraldehyde 0.0092 mmHg/μl, and 0.0046 mmHg/μl for control eyes. Figure 3 shows the curves of the relation pressure volume between diferents crosslinkers.

### Endothelial cell damage

The cell damage was evaluated with alizarin red who stain the injured cells and the intercellular spaces. With this criteria each photograph was analyzed in all groups. In the Genipin group the endothelial cell damage was 9.4±1.5%, UV riboflavin was 10.35±1.7%, Glutaraldehyde group was 40.16± 6.23%, and control was 5.91±1.8%.

**Figure 4:**
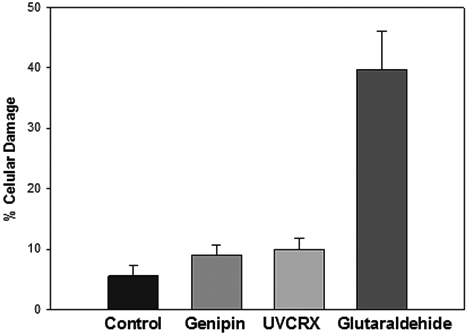
Endothelial change with different crosslinkers.

There was no difference between Genipin versus UV-CLX versus Control (Figure 4).

### Cells by mm^2^

This area was used to analyze the amount of cells destroyed by each crosslinker.

The numbers of cells by mm^2^ were genipin 2,787±80 cells/mm^2^, UV riboflavin 2,526±109 cells/mm^2^, Glutaraldehide 2,108±153 cells /mm^2^, and control 2,790±50 cells/mm^2^.

There was significant difference between genipin versus UV riboflavin (p 0.008) and between genipin, UV riboflavin versus glutaraldehyde.

### Cellular area

Cellular area studies the morphology of cells in the different crosslinkers groups. In genipin group were 277±8 µm^2^/cells, UV riboflavin 296±9 µm^2^/cell, glutaraldehyde 326±16.6 µm^2^/cell, and control 288±7 µm^2^/cell.

**Figure 5:**
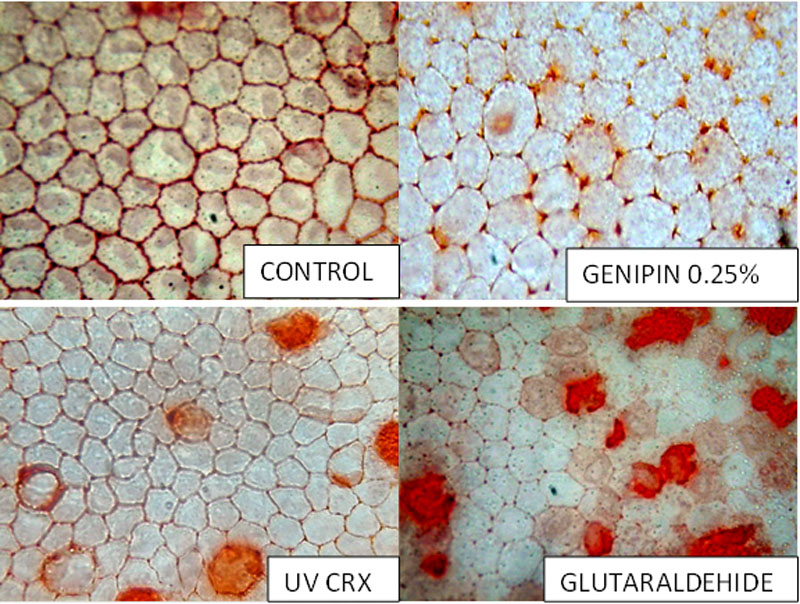
Histological Effect in corneal endothelium with different crosslinkers.

In morphological analysis occasional cells with edema and with granules were observed in the UV riboflavin group (Figure 5) and cellular lysis was observed in the glutaraldehyde group.

**Figure 6:**
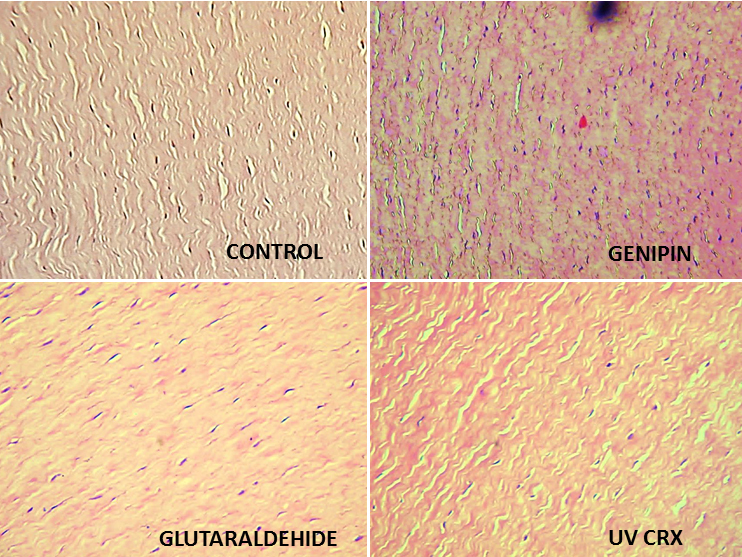
Keratocyte change with different crosslinkers.

A reduction of keratocytes was observed in the UVCRX group (40±5 cells by field) in comparison with Control (70±10 cells by field), genipin (72±8 cells by field) and glutaraldehyde (64±9 cells by field; Figure 6).

## Discussion

Corneal crosslinking have emerged as a novel strategy in the management of corneal ectasia, keratoconous, and ectasia post lasik. Currently UV-CLX have improved corneal rigidity thus reducing the progression of the disease but UV toxicity and long-term effects not only to the cornea but also in the lens and retina are still questionable, so approachs that avoid UV are required.

This study compared the result of diferents crosslinkers as well as the cytotoxic effects in the endothelial cells. The crosslinking effect in the whole eye model is similar between genipin and UV-CLX under physiologic conditions . Maximal effect is obtained with glutaraldehide the gold standard for crosslinking in vitro unfortunately the most toxical for all cells. In this study we compared the crosslinkers with a methodology, quite more similar to a in vivo situation to determinate the changes in the ocular rigidity.

Genipin, a natural compound with crosslinker properties in the cornea, showed a little more of increase in ocular rigidity and their effect was similar in corneas with or without epithelium (data not shown) with similar biomechanical effects, this could be benefical since epithelial deffects can induce more pain in the post operative and increases the risk of infection so a crosslinking system with a intact epithelium is ideal as with minimal toxicity in comparison with the UV CRX crosslinking method.

The cytotoxic effect in the endothelial cells is similar between UVCRX and Genipin in percent of cell damage (10.3% versus 9.4%) but was superior to control corneas 5.9%, with no diference in cell damage between the different treatments but there was a slightly difference between the cellular area between treatments with a increase of the area in UV-CLX, this changes could be due to the UV energy.

In corneal keratocytes a reduction of the cells were observed in the UVCRX group, this effect have been demonstrated previously in other studies [8].

In order for genipin to be therapeutically viable needs to shown an acceptable biocompatibility profile, a concern could be the effect of the genipin in the endothelial cells, morphological changes such as cytoplasmatic foaminess and vacuole formation observed occasionally in the UVCLX group could reflect temporal changes of the stress to these cells that can recover from this radiation [9].

These effects were not observed in the genipin treated corneas, as observed endothelial cells have lower area in genipin treated cornea in comparison to UVCRX, reflecting less swelling of this endothelial cells, in fact genipin have showed a protective and anti apoptotic effect by the inhibition of the NF-KappaB in hepatic cells [10], this mechanism in described in the induction of apoptosis in corneal endothelial cells [11]. Also genipin showed a protection of the retinal ganglion cells in oxidative stress models [12] other mecanism in the damage of the corneal endothelium in pathologies as a Fuchs endothelial corneal distrophy [13], so one might consider that the toxical effects to endothelium is minimal and could consider that could be protective for the endothelium, despite that this was not the objective of this study we found less toxicity in the genipin treated corneas as showed in this study.

In conclusion, we found that Genipin was similar to UV-CLX in the crosslinking of porcine corneas in the model of whole eye. However the Genipin could be applied to corneas with intact epithelium and their effect to the endothelium is minimal deserving more studies.
